# A community experiment with fully open and published peer review

**DOI:** 10.1186/1745-6150-1-1

**Published:** 2006-01-31

**Authors:** Eugene V Koonin, Laura F Landweber, David J Lipman

**Affiliations:** 1 National Center for Biotechnology Information, National Library of Medicine, National Institutes of Health, Bethesda, MD 20894, USA.; 2 Department of Ecology and Evolutionary Biology, Princeton University, Princeton, NJ 08544, USA.

We are pleased to announce a new open access journal, *Biology Direct*, which will be published online by BioMed Central. *Biology Direct *is launching with publications in the fields of Systems Biology, Computational Biology, and Evolutionary Biology, with an Immunology section to follow soon. Eventually, the journal will expand to cover other areas of biology. Launching a new research journal in biology in the year 2006 takes a lot of hubris...and/or a clearly defined goal. The crucial open access niche has been taken by the highly successful and still proliferating BMC and PLoS journals, so a new journal hardly would stand a chance and be worth the efforts of the editors and the publisher unless it defines itself in a fundamentally new way. Thus, our goals with this new journal, *Biology Direct*, are unapologetically ambitious: to establish a new, perhaps, better system of peer review and, in the process, bolster productive scientific debate, and provide scientists with useful guides to the literature.

The general view of the current system of peer review of scientific work boils down, more or less, to the tired Churchill quote on democracy: it is the worst system imaginable except for all others. Yet, all publishing scientists are painfully aware of the growing problems of the peer-review system. The crucial feature of the present peer review approach is that it is, predominantly, anonymous, and a reviewer, safe behind the veil of anonymity, has virtually absolute power over the helpless author. And absolute power we know only too well can corrupt absolutely. Hence the biased reviews, the inexplicably delayed perfunctory reviews, the pedantic reviews making a huge deal of minor quibbles, and other kinds of unfair and upsetting reviews that we all dread reading but, unfortunately, may even find ourselves writing. Of course, it is not some sinister villains who produce these obnoxious reviews, it is we peers, we distinguished members of the active scientific community. Indeed it would not be much of an exaggeration to state that all of us, on one occasion and another, have been on the receiving end of peer review abuses, and (almost) any frequent referee has probably been an abuser as well, whether accidentally, intentionally, or at least in the eye of the author.

Why is anonymity in peer review necessary despite the inevitable abuse it spawns? The answer is obvious: because there would be no truly critical reviews (or very few and far between) if the referees had to sign them. But let us ask a less trivial question: why is that? Are we always afraid of criticizing each other and getting into a serious debate? Why, no! Anyone who ever attended a scientific conference worth its salt knows that the discussions can be quite vigorous, often enough going to bare knuckles, especially during the coffee breaks or at the bar, but also in the conference room itself. Sometimes someone gets upset or offended but it is, definitely, an exception. And how priceless these discussions often are in providing us with new perspectives and fresh ideas for our research! In large part, it is for the discussion, not so much for the talks, that we go to the trouble of traveling to all these conferences, enduring the separation from our labs and families. So here is the paradox: the very same scientists – we-, who are open, constructive and, often, sharply critical in face-to-face discussions at meetings and seminars, need the protection of anonymity (and may even go to some pains to avoid hinting at one's identity) when reviewing a colleague's manuscript. The solution is quite straightforward: most of the time, a negative review or even one calling for a major revision is a hard blow to the author because it leads to rejection, the need to modify the manuscript for another (typically, lower-ranking) journal, inevitable delay with the publication for months, and, possibly, challenges for funding.

So why are scientific papers denied publication in the first place? There are two very different, major reasons: i) publication costs and space force publishers to be selective and ii) if everything anyone submits is published, it would be practically impossible to navigate the sea of publications. The Internet era, essentially, eliminates the first problem: pretty much anything can be made public at a negligible cost. Of course, there can be no free lunch, so the second problem would be exacerbated: unchecked publication would overwhelm the scientist and threaten to bury him/her under an avalanche of information (and noise).

In *Biology Direct*, we seek to live by the realities of the 21^st ^century while addressing the issue of information overflow in a constructive fashion and offering a remedy for the ills of anonymous peer review. The journal will publish "essentially anything", even papers that receive three unanimously negative reviews, the only conditions being that three Editorial Board members agree to review (or solicit a review for) the manuscript and that the work qualifies as scientific (not pseudoscientific as is the case for intelligent design or creationism) – and, of course, that the author wants his/her paper published alongside the reviews it receives. Everything in *Biology Direct *will be completely in the open: the author will invite the referees without any mediation by the Editors or Publisher, and the reviews will be signed and published together with the article. The idea is that any manuscript, even a seriously flawed one, that is interesting enough for three respected scientists to invest their time in reading and reviewing will do more good than harm if published – along with candid reviews written by those scientists. Under the *Biology Direct *rules, an author is free to solicit as many members of the Editorial Board as s/he has patience for. The philosophy behind this approach is that what really matters is not how many scientists are uninterested in a paper (or even assess it negatively, which could be the underlying reason for declining to review) but that there are some qualified members of the scientific community who do find it worthy of attention. A manuscript will be, effectively, rejected only after the author gives up on finding three reviewers or exhausts the entire Editorial Board. We believe this is fair under the rationale that work that fails, after a reasonable effort from the author, to attract three reviewers is probably of no substantial interest, even if technically solid. (While there is an opportunity to request one of the Editors to nominate outside referees in cases when the author can credibly claim that there are no Board members qualified to handle the work, we expect such cases to be rare).

Hopefully, the published and signed reviews will serve as beacons in the information sea and will alert the reader to articles of special interest, but also to potential problems in any article, and to articles where the main conclusions themselves are suspect. These are our immediate aims with *Biology Direct*. The ulterior goal, though, is a loftier one. Those of us who are old enough to have read conference proceedings books from the 1960s and 1970s will nostalgically recall the transcripts of discussions that accompanied the articles; usually these made the best reading in the book. Such discussions still occasionally appear in the *Philosophical Transactions of the Royal Society *but, generally, we do not see many of them anymore: in the 30 years elapsed since those halcyon days, biology has matured a lot but, in the process, the freewheeling spirit of debate has somehow wilted. The obvious merits of scientific maturity notwithstanding, we all seem to be poorer for this decline of published discussion, and it is our fond hope that *Biology Direct *will help to revive it.

The caveats to and dangers of the *Biology Direct *concept are many and substantial, and we are certainly aware of them. It is easy to argue that giving up strict publication criteria eliminates the competition for a place in top journals which, in part, drives the progress of research and promotion these days. In response, one might question the healthiness of this competition but, regardless, the *Biology Direct *approach, even under the best case scenario, is intended to complement, but by no means to replace, the current system. It is quite likely that both mediocre papers and outright wrong ones will creep into *Biology Direct*, and in some cases, the reviews would not identify them clearly; but then, again, can any conventional journal claim it is free of such publications? On the bright side, we believe that there is a good chance that *Biology Direct *will give the light of day to truly innovative, bold (sometimes, partly, speculative) research which, as we all know, can be extremely hard to get into high-profile journals. Perhaps the most pertinent danger is that *Biology Direct *devolves into a self-serving club where a group of scientists rotates as authors and referees. The rules have certain safeguards against developing such a mafia, by limiting the number of times any two individuals may appear as an author-reviewer pair. More fundamentally, however, everything depends on the size of the club – should it include a large group of top scientists, "clubbiness" might not be such a bad thing after all, and will make for good reading. So far, we have been enormously encouraged by the positive response of a considerable number of excellent researchers who accepted our invitation to join the Editorial Board of *Biology Direct *(see ). Clearly, this is not enough for the ultimate success of the new journal – the Board must grow substantially without losing quality. Above all, this is a community experiment in seeking new ways of communicating and discussing science, which, eventually, might have measurable effect on how we actually do science. If we, as a community, have the collective will to make it work, it will. Welcome to *Biology Direct*!

